# Superposition of vortex beams generated by polarization conversion in uniaxial crystals

**DOI:** 10.1038/s41598-022-12223-3

**Published:** 2022-05-17

**Authors:** Alexandru Craciun, Oana-Valeria Grigore

**Affiliations:** 1 Laboratory of Solid-State Quantum Electronics, National Institute for Laser, Plasma and Radiation Physics, 077125 Magurele, Ilfov Romania; 2 Doctoral School of Physics, University of Bucharest, 077125 Magurele, Ilfov Romania

**Keywords:** Engineering, Optics and photonics, Physics

## Abstract

An optical system comprising a *c*-cut uniaxial crystal positioned between two axicons and illuminated by a Gaussian or a Laguerre–Gauss mode was used to demonstrate the generation of various vector vortex beams. We focused the generated beams using a 1 m focal length lens and we investigated their intensity profile and the polarization state in the focal plane of the focusing element. We showed that the achieved intensity profile can be controlled by changing the polarization state of the beam incident on the crystal. We observed that, for a particular configuration of the system, the initial circular symmetry of the beam is no longer preserved. The experiments were performed with Ti:Sapphire lasers that were operated in continuous wave mode, as well as in pulsed regime. The optical system presented here ensures a convenient way to generate a great variety of vector vortex beams and it is expected to be of interest for applications that use low and high-power laser sources, such as STED microscopy, light trapping, or material processing.

## Introduction

Structured light is referring to the configurations of the electromagnetic field with non-trivial variations in phase, amplitude, or polarization state^1,2^. Particular types of structured light fields are vortex beams. They are characterized by a doughnut (or ring-like) transversal intensity profile with zero intensity in the centre and a phase that changes by an integer multiple *m* of 2*π* along a contour around that zero-intensity point. The phase structure enables the beam to carry an amount *mħ* of orbital angular momentum (OAM) per photon, where *m* is referred to as topological charge and *ħ* represents the reduced Planck's constant. The polarization state of such beams is homogeneous. If the polarization state is space-variant across the transversal section, the vortex beam is commonly named vector vortex beam (VVB). This is usually described as a collinear superposition of two complex conjugated vortex modes with orthogonal polarization states. On the other hand, the superposition of multiple modes that are not complex conjugated generates a so-called hybrid vector beam (HVB), which has some interesting properties not observed in the case of VVBs^3^. VVBs are increasingly more relevant for today’s emerging fields such as STED microscopy^4^, quantum comunication^5^, material processing^6,7^, or optical manipulation^8^.

Due to the remarkable characteristics of VVBs and HVBs but also to their considerable applicability potential, plenty of generation techniques has been proposed. Some of them regard the VVBs generation by direct emission of laser beams with the desired optical vortex phase inside a laser cavity^9–11^. Despite the advantages of these techniques, like low cost, high power, and high efficiency, the systems could be deemed rigid in terms of the assortment of the generated VVBs. Other methods make use of specially designed optical elements with spatially variant polarization properties placed outside of the laser cavities. Such elements are patterned liquid crystal retarders (q-plates)^12^, birefringent plates written with sub-wavelength gratings^13^, metasurfaces^14^, Brewster angle reflectors^15^ or anisotropic films^16^. Some alternative methods make use of interferometric optical systems that combine two beams with orthogonal polarization states generated either using spiral phase plates (SPP)^17^ or spatial light modulators (SLM)^18^. Method such as that described by Liu et al.^18^ can be also used for the generation of HVB as the phase can be independently controlled for each arm of the interferometer. However, SLM-s are characterized by low efficiency due to diffraction losses. The approaches using combinations of diffractive or geometrical optical elements are much simpler and more efficient^19,20^. Quiceno-Moreno et al.^21^ used two q-plates placed in line, one having tunable retardance, for the generation and the investigation of HVBs in both near and far-field.

Polarization conversion that is due to the propagation of a beam along the anisotropy axis of a uniaxial crystal could also be an alternative to the methods mentioned above. The possibility to generate vortex beams relying on polarization conversion in *c*-cut crystals was explored in references^22–27^. In the experimental works, the uniaxial crystal was illuminated either by Gaussian^26^ or Bessel beams^28,29^. The advantage of using Bessel beams is given by the possibility to achieve any value of polarization conversion efficiency up to 100% compared to the case of Gaussian beams where the maximum efficiency conversion is 50%. To assist practical applications we aimed to develop a simple, reliable, and efficient system that could be easily adapted to any laser source to achieve a variety of vortex beams mixtures.

In most of the studies that describe the dynamics of polarization conversion in uniaxial crystals, the techniques rely on focusing the input laser beam inside the uniaxial crystal or close to its surface. However, this method makes almost impossible the use of high-power light sources. The paper of Shvedov et al.^26^ is the only one that describes an optical system illuminated by a divergent laser beam, but only for the generation of single- and double-charge vortex beams. It is emphasized that in this way the possibility of self-focusing is avoided and thus this method is appropriate for femtosecond (fs) high power laser applications, while also being cheaper and less sensitive to wavelength variation than using diffractive optics. Despite these advantages, the method is limited by the polarization conversion efficiency. Loussert et al.^30^ have demonstrated that 100% efficiency can be achieved using a single piece of uniaxial material illuminated by a nearly conical superposition of plane-waves. In addition, a collimated input beam on an optical system consisting of an SPP and a uniaxial crystal with a special shape has been theoretically proven to generate HVBs^31^. Using uniaxial crystals to generate VVBs and HVBs may have some advantages over the other methods, for instance, the possibility to achieve conversion without losses and also to use high power fs pulsed beams. However, in most of the aforementioned research regarding polarization conversion, the exploration of the polarization state and the intensity profile was done for a collimated beam. The effects of subsequent focusing of the beam have not been explored.

In this study, we investigated, in the focal plane of a lens, the polarization state and the transverse intensity distribution of VVBs or HVBs. Such beams are produced as a result of the overlapping of the vortex modes generated by an optical system comprising a Sapphire *c*-cut uniaxial crystal placed between two axicons and illuminated by a Gaussian or a Laguerre–Gauss mode beam. The advantages of using this system are given by: (i) yielding a conversion efficiency of up to 100% in relation to the thickness of the uniaxial crystal and the features of the axicons; (ii) achieving of VVBs and HVBs by varying the input beam characteristics like spatial distribution and polarization; (iii) can be used for a wide range of beam power conditions. The Sapphire crystal was chosen because of its hardness and ability to withstand mechanical and thermal shocks compared to other used crystals, such as Calcite, or potassium dihydrogen phosphate (KDP) that, in addition, are hygroscopic; furthermore, the Sapphire crystal could be fabricated at large enough dimensions. We theoretically describe the phenomenon of polarization conversion in the *c*-cut uniaxial crystal using Jones matrices and we simulate the beam propagation through the system. One of the most intriguing phenomena that have been observed for the linearly polarized Laguerre–Gauss beam incident on the optical system is the breaking of the circular symmetry as the beam propagates to the lens’s focal plane. This behaviour is theoretically explained in one of our previous work^31^ as well as in Quiceno-Moreno et al.^21^ to be caused by the Gouy phase shift associated with the propagation of Laguerre–Gauss modes to the focal plane of a lens.

## Results

### Generation of VVBs and HVBs using c-cut uniaxial crystals

In this section, we will briefly discuss the generation of VVBs and HVBs by polarization conversion in uniaxial crystals. Let's consider a *c*-cut uniaxial crystal (i.e., with the crystal's anisotropy axis perpendicular to the plane of the transparent surfaces) oriented with the crystal axis parallel to the propagation direction of the beam (optical axis). It is well known that a plane-wave passing through a uniaxial material will experience: (i) an ordinary refractive index*,* ${n}_{o}$, if the initial direction of the electric field before the crystal is perpendicular to the crystal axis, in this case, the polarization state is azimuthal; or (ii) an extraordinary refractive index, ${n}_{e}$*,* if the initial direction of the electric field before the crystal is parallel to the plane defined by the plane-wave's propagation direction and the crystal axis, and in this case, the polarization state could be considered radial. Otherwise, the initial polarization will not be preserved during the propagation of the beam through the uniaxial crystal. The change of polarization is determined by the phase difference acquired between the ordinary and the extraordinary waves and by the orientation of the extraordinary axis.

**Figure. 1:**
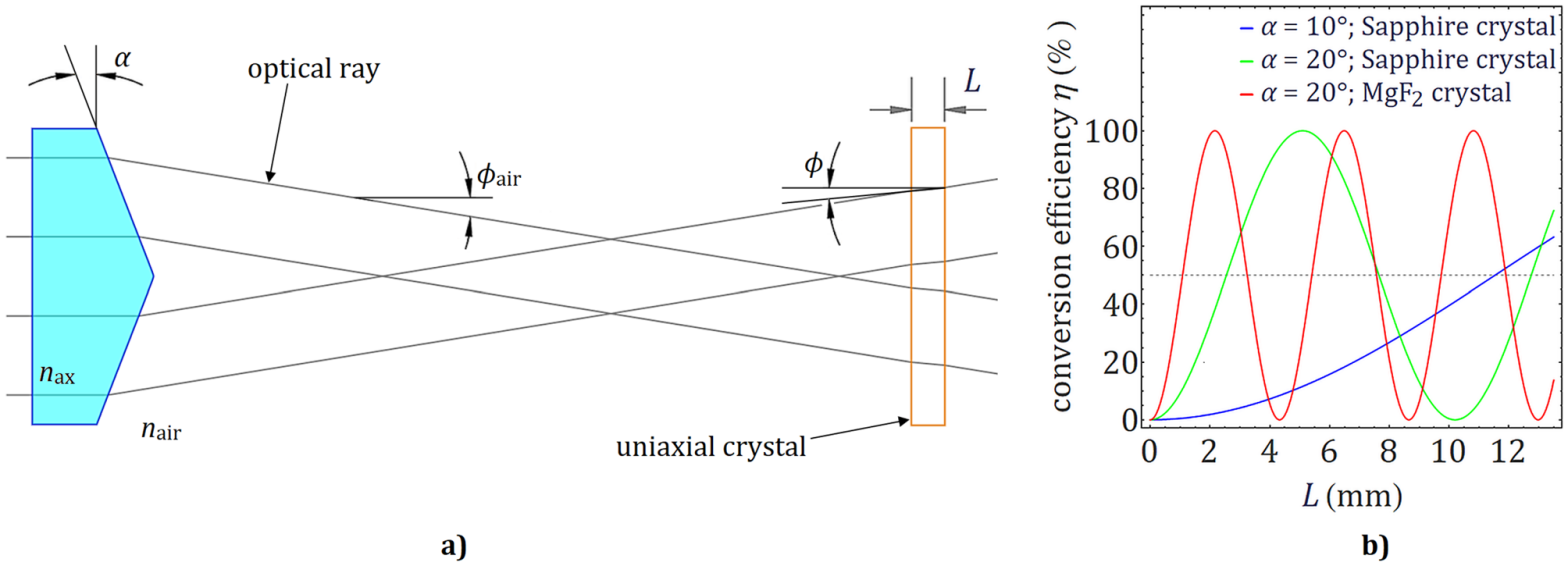
(**a**) Ray-tracing representation through the first axicon and the uniaxial crystal that shows the connotation of the terms like the length of the crystal *L*, the physical angle of the axicon α, the tilt angle with of the beam in free space *ϕ*_*air*_, and inside the crystal, *ϕ*, with respect to the optical axis. (**b**) The conversion efficiency of the fundamental polarization mode as a function of the crystal type and thickness for given values of the physical angle of the axicon, α. The MgF_2_ crystal was considered only as an example to show that its characteristics influence the conversion efficiency of the polarization differently from the Sapphire crystal.

Our optical system comprises a part made up of two axicons with a *c*-cut uniaxial crystal placed between them. This part solely will be discussed in this section. The first axicon is used to produce a divergent beam with a conical wavefront incident on the crystal. The tilt angle of the rays inside the crystal (see Fig. 1a) is related to the characteristics of both the axicon and the crystal, and is given by the solution of the system of equations below (Eq. 1):

1 $$\left\{\begin{array}{l}{n}_{\mathrm{ax}}\mathrm{sin}\alpha ={n}_{\mathrm{air}}\mathrm{sin}\left(\alpha +{\phi }_{\mathrm{air}}\right)\\ {n}_{\mathrm{air}}\mathrm{sin}{\phi }_{\mathrm{air}}=\frac{{n}_{e}\left(\phi \right)+{n}_{o}}{2}\mathrm{sin}\phi \end{array}\right.$$

These equations arise from the application of Snell’s law at the interface between the first axicon and air (rear surface), and the interface between air and the uniaxial crystal, considering a ray that is initially parallel to the optical axis. Herein n_ax_ is the refractive index of the axicon, α is the physical angle of the axicon, n_air_ is the refractive index of air, ${\phi }_{\mathrm{air}}$ and $\phi$ are the tilt angles of the rays in the air due to the axicon, and inside the crystal respectively, and ${(n}_{e}\left(\phi \right)+{n}_{o})/2$ is an average refractive index for the uniaxial crystal where ${n}_{e}\left(\phi \right)$ is the effective extraordinary refractive index that depends on the tilt angle $\phi$ with respect to the crystal axis.

The beam resulting after the refraction from the first axicon is a circular superposition of plane-waves having a fixed tilt with respect to the optical axis; hence, all of the waves experience the same extraordinary refractive index. In this particular configuration, the orientation of the direction of extraordinary polarization is always radially with regard to the optical axis. As a consequence of this feature, the change in polarization is accompanied by a transverse mode conversion into a vortex mode. The amount of OAM resulting from this transition balances the change of spin angular momentum (SAM) due to the polarization conversion. This phenomenon is known as optical spin-orbital angular momentum coupling or polarization conversion in uniaxial crystals. The second axicon is used to recollimate the beam.

Let’s consider in the following that an optical beam with linear polarization passes through the optical system. The propagation through the uniaxial crystal changes the polarization, intensity, and phase of the beam. The ordinary and the effective extraordinary refractive indexes are very similar, which means that is not a significant spatial walk-off between the waves. In this instance, we can consider that the intensity profile of both ordinary/extraordinary waves changes in an identical manner with the propagation through the crystal. To find a simplified mathematical description of the investigated phenomenon, we should factor out the radial dependence. We will keep only the azimuthal dependence of the phase. In this case, we can associate the crystal with an axially rotated waveplate, although the transformation produced is much more complex. However, for the numerical simulations, we have considered the effect of the propagation through the crystal on the intensity, phase, and polarization of the beam.

Linear polarization could be described as a linear combination of two orthogonal circularly polarized states $\left|+\rangle , \right|-\rangle$ with equal weights; however, the phase difference between the circularly polarized components gives the orientation of the polarization plane. We can also use two orthogonally linearly polarized states to describe the radially respective azimuthally polarization states $\left|\mathrm{R} \rangle \; \mathrm{and} \; \right| \mathrm{A}\rangle$. These states are regarded as the modal beams with eigen-polarization^32^ in uniaxial crystals since they have a well-defined refractive index; therefore they do not change their structure up to the scale transformation due to diffraction. The refractive index experienced by the radial mode is the effective extraordinary refractive index ${n}_{e}\left(\phi \right)$, while the azimuthally mode experiences the ordinary refractive index ${n}_{o}$. The transition from the basis of radial/azimuthal states to the basis generated by the linear polarization states can be achieved via a rotation matrix, which depends on the azimuthal angle of rotation around the optical axis $\theta$, measured counterclockwise from the horizontal axis. Equation (2) gives the connection between an arbitrary Jones vector written in a circular basis respective radial/azimuthal basis:

2 $$\left(\begin{array}{c}| + \rangle \\ |\boldsymbol{ }- \rangle \end{array}\right)=\frac{1}{\sqrt{2}}\left(\begin{array}{cc}1& -\mathrm{i}\\ 1& \mathrm{i}\end{array}\right)\left(\begin{array}{cc}{\cos}\theta & -\mathrm{sin}\theta \\ \mathrm{sin}\theta & {\cos}\theta \end{array}\right)\left(\begin{array}{c}| \mathrm{R} \rangle \\ |\boldsymbol{ }\mathrm{A} \rangle \end{array}\right)={\mathbb{M}}\left(\theta \right)\left(\begin{array}{c}| \mathrm{R} \rangle \\ |\boldsymbol{ }\mathrm{A}\rangle \end{array}\right)$$

We would like to point out that the following analysis is proper for plane-waves, but it is also valid for rays. As long as the beam is convergent/divergent, we can associate a ray having the same direction as each of the plane-waves that form the beam. In the small birefringence regime and for the propagation of the beam close to the optical axis, we can trace the intensity of the beam along the ray. The phase changes will be determined by the optical path length between the input and the output planes. For the radially and azimuthally polarized modes we have well determined refractive indexes and we can easily model the propagation through the crystal as a multiplication by a phase factor, as given in Eq. (3).

3 $${\left(\begin{array}{c}| \mathrm{R} \rangle \\ |\boldsymbol{ }\mathrm{A} \rangle \end{array}\right)}_{\mathrm{out}}=\left(\begin{array}{ll}{e}^{\mathrm{i} {k}_{0} {n}_{e}\left(\phi \right) \frac{L}{{\cos}\phi }}& 0\\ 0& {e}^{\mathrm{i} {k}_{0} {n}_{o} \frac{L}{{\cos}\phi }}\end{array}\right){\left(\begin{array}{c}| \mathrm{R} \rangle \\ |\boldsymbol{ }\mathrm{A} \rangle \end{array}\right)}_{\mathrm{in}}$$

By replacing the expression for the amplitude of the radial/ azimuthal polarized modes as given in Eq. (2), we find:

4 $${\left(\begin{array}{c}| \mathrm{R} \rangle \\ |\boldsymbol{ }\mathrm{A} \rangle \end{array}\right)}_{\mathrm{out}}={\mathbb{M}}^{-1}\left(\theta \right){\left(\begin{array}{l}| + \rangle \\ |\boldsymbol{ }- \rangle \end{array}\right)}_{\mathrm{out}}=\left(\begin{array}{ll}{e}^{\mathrm{i} {k}_{0} {n}_{e}\left(\phi \right) \frac{L}{\mathrm{cos}\phi }}& 0\\ 0& {e}^{\mathrm{i} {k}_{0} {n}_{o} \frac{L}{\mathrm{cos}\phi }}\end{array}\right){\mathbb{M}}^{-1}\left(\theta \right){\left(\begin{array}{l}| + \rangle \\ |\boldsymbol{ }- \rangle \end{array}\right)}_{\mathrm{in}}$$

The polarization state of the exit beam expressed using right/left circularly polarized states results by applying the matrix ${\mathbb{M}}$ over Eq. (4). In Eq. (5) we have a concise expression, using the Jones matrix ${\mathbb{U}}$, for the effect of the crystal on the incident field. This matrix depends on the phase difference $\Phi$ between the ordinary and the extraordinary waves, its expression being given in Eq. (6).

5 $$\begin{aligned} {\left(\begin{array}{c}| + \rangle \\ |\boldsymbol{ }- \rangle \end{array}\right)}_{\mathrm{out}} & ={\mathbb{M}}\left(\theta \right)\left(\begin{array}{cc}{e}^{\mathrm{i} {k}_{0} {n}_{e}\left(\phi \right) \frac{L}{\mathrm{cos}\phi }}& 0\\ 0& {e}^{\mathrm{i} {k}_{0} {n}_{o} \frac{L}{\mathrm{cos}\phi }}\end{array}\right){\mathbb{M}}^{-1}\left(\theta \right){\left(\begin{array}{c}| + \rangle \\ |\boldsymbol{ }- \rangle \end{array}\right)}_{\mathrm{in}} \\ & =\frac{{e}^{\mathrm{i} {k}_{0} \left[{n}_{e}\left(\phi \right)+{n}_{o}\right] \frac{L}{2\mathrm{ cos}\phi }}}{2}\left(\begin{array}{cc}\mathrm{cos}\frac{\Phi}{2}& \mathrm{i} {e}^{-2 \mathrm{i} \theta }\mathrm{sin}\frac{\Phi}{2}\\ \mathrm{i} {e}^{2 \mathrm{i }\theta }\mathrm{sin}\frac{\Phi}{2}& \mathrm{cos}\frac{\Phi}{2}\end{array}\right){\left(\begin{array}{c}| + \rangle \\ |- \rangle \end{array}\right)}_{\mathrm{in}} \\ &= {e}^{\mathrm{i} {k}_{0} \left[{n}_{e}\left(\phi \right)+{n}_{o}\right] \frac{L}{2 \mathrm{cos}\phi }}\left(\mathrm{cos}\frac{\Phi}{2} {\mathbb{I}}+\mathrm{i sin}\frac{\Phi}{2} {\mathbb{T}}(2 \theta )\right){\left(\begin{array}{c}| + \rangle \\ |- \rangle \end{array}\right)}_{\mathrm{in}}={\mathbb{U}}\left({\Phi}{,}2\theta \right){\left(\begin{array}{c}| + \rangle \\ |- \rangle \end{array}\right)}_{\mathrm{in}} \end{aligned}$$

$\Phi$ is defined as:

6 $$\Phi=\frac{2\pi }{\lambda }\frac{L}{\mathrm{cos}\phi }\left[{n}_{o}-{n}_{e}\left(\phi \right)\right]$$

The matrix ${\mathbb{U}}$ from Eq. (5) can be written as a weighted sum of the matrices ${\mathbb{I}}$ and ${\mathbb{T}}$, multiplied by a phase factor, where ${\mathbb{I}}$ is the identity matrix. Its presence in the summation conveys that there is a part of the initial beam that does not change its initial polarization state. The weight of the mode preserving the polarization state of the initial beam is proportional to ${\cos}\Phi/2$. On the other hand, the matrix ${\mathbb{T}}$ is antidiagonal and incorporates on the first row the spatial phase term $\mathrm{exp}\left(-2\mathrm{ i }\theta \right)$ and on the second row the term $\mathrm{exp}\left(2\mathrm{ i }\theta \right)$. This matrix transforms the right circularly polarized mode into a left circularly polarized mode while adding a vortex spatial phase with topological charge + 2, and also transforms the left circularly polarized mode into a right circularly polarized mode while adding a vortex spatial phase with topological charge − 2. This transformation preserves the total amount of optical angular momentum. The phase difference $\Phi$ is proportional to the distance travelled by the beam through the crystal. Therefore, the contribution to the generated beam of the part preserving the initial polarization state varies cosinusoidal as the beam propagates through the crystal, losing energy to the vortex counterpart, which has an orthogonal polarization state. This is caused by the interference between the ordinary and the extraordinary waves as the phase difference between those waves changes with the propagation distance. The conversion efficiency is the fraction of energy that goes to the newly generated modes; in other words, it is the squared amplitude of the coefficient in front of the matrix ${\mathbb{T}}$ in Eq. (5). The conversion efficiency formula is given in Eq. (7).

7 $$\eta ={\mathrm{sin}}^{2}\frac{\Phi}{2}$$

For given values of the ordinary, ${n}_{o},$ and extraordinary, ${n}_{e},$ refractive indexes, crystal thickness *L*, wavelength *λ,* and rays tilt inside the uniaxial crystal $\phi$, the effect of the crystal on the phase and polarization of the beam is like that of an axially rotated waveplate of a certain type. For example, when $\Phi$ = *π*, the crystal matches the half-waveplate condition and the polarization conversion efficiency is *η* = 100%. In this case, the resulting beam at the output of the crystal has an annular transversal intensity profile with a polarization state that depends on the input polarization. The initial right-circularly polarized light will be transformed into left-handed circular; conversely, if the initial polarization is left-circularly, then the generated mode will be right-circularly polarized. The conservation of the angular momenta determines that a vortex mode with a double topological charge, $\left|m\right|=2$, with the sign opposite to the transferred SAM, will be generated. For non-circular input polarization, the beam generated by the uniaxial crystal will be a mixture of two vortex modes with topological charges *m* =  ± 2 having an inhomogeneous polarization state. The amount of energy that is converted will be determined by the thickness of the crystal *L* and the tilt angle, $\phi ,$ inside the crystal. The conversion efficiency of the initial polarization mode as a function of the crystal type and thickness for a given value of $\alpha$, is shown in Fig. 1b. That part of the laser mode whose polarization has not been converted will propagate along the same optical axis together with the newly generated modes.

By adding an SPP in the system, which will change the topological charge of the composing modes by 1 or − 1, we can achieve the vortex mode composition ± 1 and 3 or ∓ 1 and − 3, respectively. In this case, the intensity profile of the resulting beam will be affected by the interference of the composing beams.

### Experimental setup

**Figure 2:**
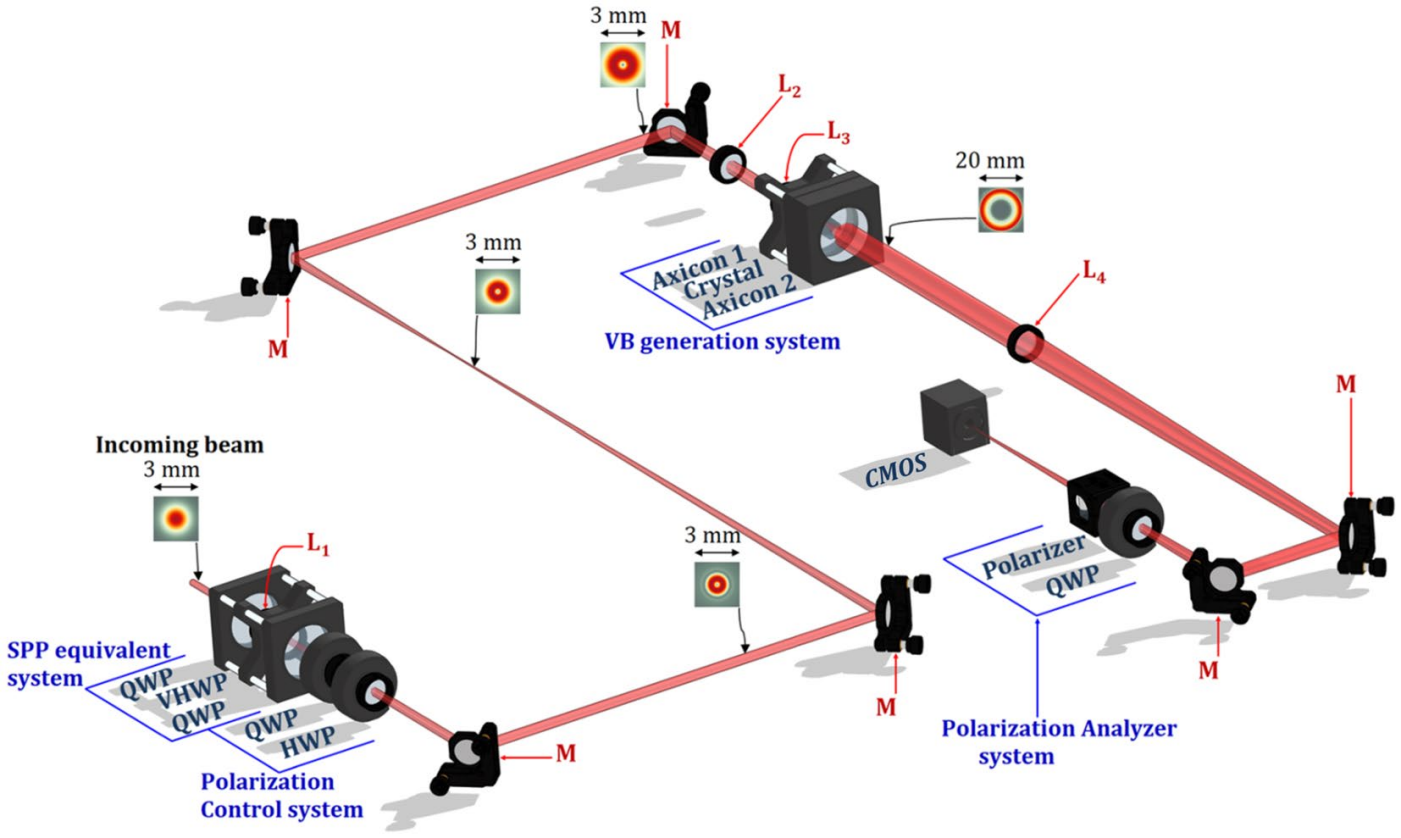
The experimental setup comprises four main parts: an SPP equivalent system consisting of two quarter-wave plates—QWP and a vortex half-wave plat—VHWP; a Polarization Control system composed of a quarter-wave plate—QWP and a half-wave plate—HWP; a Vector Vortex Beam generation system which contains two fused silica ultra-quality axicon lenses with physical angle of 20°—Axicon 1 and Axicon 2 and a uniaxial Crystal, and a Polarization Analyzer system made up of a quarter-wave plate—QWP and a Polarizer. The mirrors *M* were used to direct the laser beam throughout the optical system up to the CMOS-video camera. L_1_, L_2_, L_3_, L_4_: lenses used in laser beam collimation and focusing processes. The focal lengths of the lenses are f_1_ = 1200 mm, f_2_ = − 75 mm, f_3_ = 200 mm, f_4_ = 1000 mm. Theoretical transverse distributions of the laser beam generated by the Ti:Sapphire oscillator, used in the experiments are indicated at different positions of the setup.

The experimental setup used to generate the superposition of vortex beams and to investigate their polarization state is presented in Fig. 2. It consists of four main parts: (i) an SPP equivalent system (SPPS) comprising a half-wave q-plate (VHWP) placed between two quarter waveplates (QWP); (ii) a Polarization Control system (PCS), composed of a QWP followed by a half waveplate (HWP), where both components rotate around the optical axis to adjust the polarization state; (iii) a Vector Vortex Beam generation system (VBS), and iv) a Polarization Analyzer system (PAS).

Experiments were performed with and without SPPS within the system in order to generate various VVBs and HVBs. Considering the presence of the SPPS, a Gaussian laser beam first passes through the SPPS which generates a vortex beam with uniform polarization and topological charge *m* =  − 1. The lens L_1_ brings the beam to the far-field where the intensity profile has the typical doughnut-like shape. The resulting uniform polarized vortex beam is expanded and collimated by the telescope formed of L_2_ and L_3_ lenses. Then, the beam illuminates the VBS, which adds or decreases the topological charge by 2, leading to a superposition of vortex beams. The weight of each vortex beam depends on the crystal thickness and on the incoming laser beam polarization that is controlled by the PCS. To investigate the polarization state of the generated VVBs and HVBs in the far-field region, intensity distributions of the beams were acquired in the focal plane of the lens L_4_ with a CMOS camera. The PAS was used to determine the polarization of the beam from multiple experimentally acquired intensity profiles.

### Numerical simulation

The numerical simulations, up to the VBS, were done considering the polarization noninteracting with the physical propagation of the beam because optical elements from the PCS change the polarization in the same way on the entire cross-section of the beam. Otherwise, the polarization state of the beam right before the first axicon is decomposed in radial/azimuthal polarization states and these modes are traced through the system considering the effective extraordinary respective ordinary refractive index for the crystal. At the output of the VBS, the two modes are recomposed and propagated to the lens L_4_. The resulting beam profiles are studied in the focal plane of the lens L_4_.

All the wave-plates, including the VHWP, are ideal components modelled using Jones matrices. The diffraction of the beam was computed using a combination of Fresnel integral for the far-field region, and the Spectrum of Plane-Waves^33^ (SPW) method for the near-field. In addition, the propagation through the optical components is evaluated numerically using geometrical optics, by tracing the intensity and phase along the ray with a correction factor for the intensity according to the change in ray density between the input and the output planes.

For the propagation of the beam to the focal plane of the lens L_4_, we have used a variation of the Debye-Wolf diffraction integral (DW). Our implementation is different from the conventional DW regarding the way we evaluate the wave-front aberrations, which are normally determined from the deviation of the wavefront from a reference sphere centred on the focal point. Having a plane wavefront before an ideal focusing element, all the rays converge to one point and the optical path length is equal for all the rays. If optical aberrations are present, we expect that the optical path length would not be the same for all rays, and the rays do not intersect at the same point. Every point source generates a spherical wave, however for a well-designed system the focal spot is much smaller than the curvature radius of such a wave; thus, we can approximate it as a plane-wave. We can associate a plane-wave to any point source on the plane tangent to the focusing element and a ray on which the plane-wave is centred. Our approach is to evaluate the wavefront aberration as the following quantity:

8 $$\Psi \left(\mathbf{k}\right)=\mathrm{exp}\left[\mathrm{i }{k}_{0} S\left(\mathbf{k}\right)+\mathrm{i }\mathbf{k}\cdot {\mathbf{r}}_{\mathrm{s}}\right],$$

**k**.

*S* denotes the optical path length along the ray starting from a plane before the focusing element and the focal plane and corresponding to the plane-wave indexed by **k**, **r**_*s*_ is the coordinate of intersection between the ray and the focal plane, and *k*_0_ = 2*π*/*λ*. The quantity Ψ(**k**) is equal to the phase acquired by a plane wave indexed by **k** between the plane tangent to the rear surface of the lens and the focal plane plus the phase acquired by the ray indexed **k** due to the propagation through the lens up to the same plane tangent to the lens.

The electric field in the focal plane is given by the equation:

9 $${\mathbf{V}}_{focus}\left(\mathbf{r}\right)=-\frac{\mathrm{i}}{\lambda }\int {\mathbb{A}}\left(\mathbf{k}\right){\mathbf{V}}_{lens}\left(-\frac{\mathbf{k}}{{k}_{z}} f\right)\frac{{k}_{0}^{2}}{{k}_{z}^{2}}\mathrm{exp}\left[\mathrm{i \Psi }\left(\mathbf{k}\right)+\mathbf{k}\cdot \mathbf{r}\right]\mathrm{d}\mathbf{k}$$

${\mathbf{V}}_{\mathrm{lens}}$ is the electric field associated with the beam, in a plane after the lens ${\mathrm{L}}_{4},$ that is tangent to the rear vertex of the lens. The spherical phase induced by the lens was factored out because it is perfectly cancelled by the effect of the propagation to the focal plane*.* The spherical phase is $\mathrm{exp}\left(-\mathrm{i }2\pi /\lambda \sqrt{{f}^{2}+{\mathrm{r}}^{2}}\right)$, where $f$ is the back focal length of the lens (distance between the rear vertex and the focal plane).

The matrix:

10 $${\mathbb{A}}\left(\mathbf{k}\right)=\left(\begin{array}{ll}1+\frac{{k}_{z}}{{k}_{0}}-\left(1-\frac{{k}_{z}}{{k}_{0}}\right)\mathrm{cos}2\theta & -\left(1-\frac{{k}_{z}}{{k}_{0}}\right)\mathrm{sin}2\theta \\ -\left(1-\frac{{k}_{z}}{{k}_{0}}\right)\mathrm{sin}2\theta & 1+\frac{{k}_{z}}{{k}_{0}}+\left(1-\frac{{k}_{z}}{{k}_{0}}\right)\mathrm{cos}2\theta \\ -2\frac{{k}_{z}}{{k}_{0}}\mathrm{cos}\theta & -2\frac{{k}_{z}}{{k}_{0}}\mathrm{sin}\theta \end{array}\right)$$

^34^, where *k*_*z*_ is the longitudinal wave-vector of the plane-wave, ${k}_{z}=\sqrt{{k}_{0}^{2}-{\mathbf{k}}^{2}},$ and *θ* is the azimuthal angle around the optical axis.

### Experimental results

In the experiments, we have used a Ti:Sapphire oscillator that was operated in continuous-wave, as well as a high-power laser system that generates pulses of up to 2 mJ. The intensity distribution of the beams generated by each laser source is of Gaussian type.

The rounded tip of the first axicon from VBS will induce aberrations in the central part of a beam up to 1 mm from the optical axis, thus we needed a reasonably large beam incident on it. For this reason, the beam from the oscillator, which has a diameter of 1.7 mm, is magnified with the help of L_2_ and L_3_ lenses. On the other hand, the diameter of the laser beam delivered by the high-power laser system is 12 mm, large enough not to be aberrated by the tip of the axicons, the reason for which the lenses have been removed when this beam was used.

The Sapphire crystals from VBS were purchased for thickness values for which the polarization conversion efficiency reaches 50% (2.65 mm) and 100% (5.3 mm), respectively. Following the measurements, it was observed that the deviation from these values is in the thickness error of 0.1 mm given by the manufacturer, which might have a small influence on the obtained results.

The weight of the composing modes of the VVBs or HVBs generated using the system shown in Fig. 2 is determined by the polarization state of the beam incident on the crystal, which in turn could be controlled by the PCS. The QWP converts a linearly polarized beam into a circular/elliptical polarized beam while HWP is used to rotate the polarization. By controlling the weight of each mode, we can control the distribution of the focal spot. Any of these VVBs and HVBs could be represented on the Poincaré sphere, associated with the Stokes parameters S_1_, S_2_, and S_3_ for the polarization state of the beam incident on the uniaxial crystal. Orthogonal beams occupy points at opposite ends of the sphere diameter. To the north and south poles are represented the VVB or the HVB corresponding to right- and left-circularly input polarization states, respectively, while any VVB or HVB along the equator line is generated by the linear polarization before the VBS. All the elliptically input polarization states will generate VVBs or HVBs with intermediary configurations.

**Figure 3:**
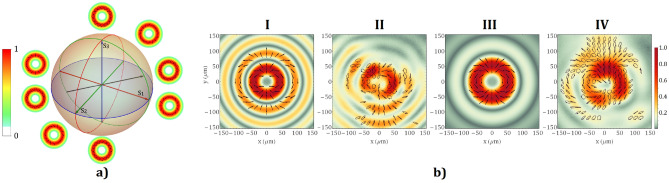
(**a**). Theoretical intensity and polarization distributions in the transverse plane for VBs with *m* =  ± 2 are represented on the Poincaré sphere. (**b**) The polarization map and the intensity distribution of the theoretical (I and III) and experimental results (II and IV) for a VBS that has a Sapphire crystal with 5.44 mm thickness and the incident beam is delivered by the oscillator (I and II) and by the high-power laser system (III and IV).

We will start by analyzing the simplest case of the polarization conversion in our system, namely for the Sapphire crystal with a thickness of 5.44 mm illuminated by a Gaussian beam. According to Fig. 1b, the polarization conversion efficiency at this point is close to maximum. Consequently, for this configuration, the resulting beam is a superposition of vortex modes with topological charges *m* =  ± 2 having variable weights. Positive or negative double charge vortex beams with homogeneous orthogonal polarization states could be generated if the polarization of the incident laser beam on the VBS is left or right-handed circularly. The transition between these two vortex beams is achieved by transforming the input circular polarization into elliptical polarization and obtaining various intermediate configurations of VVBs. The energy contained in one of the vortex modes decreases, down to zero value, by the same amount as it increases in the other mode. The intensity distribution of the VVBs in the focal plane of the lens L_4_ is given by the sum of the intensities of the composing modes. Each VVB has an elliptical polarization state with the same handedness across the whole aperture, given by the most intense generated mode, and with the orientation of the ellipse rotating linearly with the azimuthal angle by 4*π* along a closed contour around the singularity. When the input laser beam is linearly polarized, the generated vortex modes have the same intensity. Hence, the superposed right- and left-handed circular polarized modes will provide an overall linear polarization state with a spatially variable direction. The intensity and polarization distribution for these cases are shown in Fig. 3a.

By calculating the Stokes parameters of the electric field, we map the distributions of spatially variant states of polarization. In Fig. 3b are represented polarization ellipses for a vertically polarized input laser beam provided by the CW Ti:Sapphire oscillator and by the high-power laser system, respectively, as well as the theoretical results for the propagation through the optical system of the Gaussian laser beam. The intensity (S_0_) is shown using false colours. The angle of orientation of the major semi-axis of the polarization ellipse and the degree of ellipticity were calculated from the Stokes parameters. Ellipses were rendered for an intensity threshold above 0.3 from the maximum value. For the simulations, the polarization state is indicated using ellipses that correspond to the electric field calculated using the formula provided in Eq. (9).

One can notice that the experimental result for the oscillator beam (Fig. 3bII) shows an intensity distribution characteristic for an *m* = 2 doughnut vortex beam. The Bessel rings in the focal plane of the lens L_4_ are due to the thin ring like-shape of the beam intensity profile incident on it. We know that the Fourier transform of the Bessel beam is an infinitely thin ring, thus we can expect that the thinner the ring-like intensity distribution in the plane of L_4_ is, the more distinguishable the Bessel rings will be. In addition, a residual conical phase will be present if the beam incident on the VBS is not perfectly collimated. This will also cause Bessel rings in the focal plane. The central singularity with *m* = 2 is split into two *m* = 1 singularities. This is most likely due to the presence of a moderate fraction of a non-vortex beam, which does not have zero intensity on the optical axis. The authors have studied the diffraction pattern generated by the illumination of an SPP with a laser beam whose wavelength is different from the design wavelength of the SPP^35^. The resulted beam is a collinear overlap between a non-vortex mode and multiple vortex modes, the most intense of which is that with the same topological charge as the SPP. Their superposition causes the split of the singularity point into a number of singularities that is equal to the topological charge of the SPP. We know for a fact that the polarization conversion efficiency is not exactly 100% for our system because the crystal thickness is not 5.3 mm, thus the residual *m* = 0 mode is present. Furthermore, the focal spot is sensitive to the alignment of the beam along the optical axis of the VBS. Thus, the small misalignments in the optical setup may contribute to this phenomenon as well. For instance, the astigmatic transformation of vortex beams is often used to analyse the order of the topological charge^36^. We know from the previously mentioned paper that the presence of astigmatism will produce a maximum intensity on the optical axis for even topological charges. For linear input beam polarization on the crystal, we expect a VVB with linear polarization as it is shown in Fig. 3bI and III. However, elliptical polarization was also observed, most likely because of the uncertainty in the alignment of the fast axis of the QWP from PCS to achieve linear input polarization or of the axis of the QWP from PAS for the measurement of the polarization.

**Figure 4:**
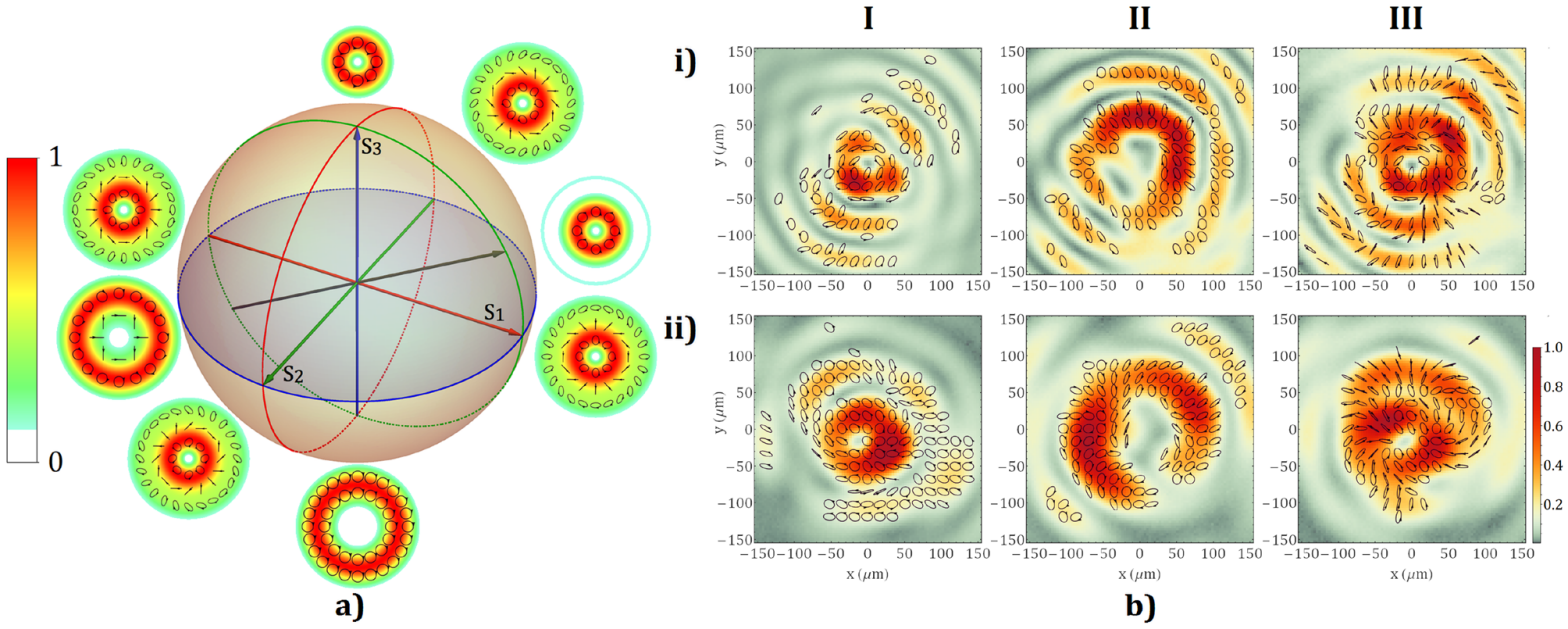
(**a**) Theoretical intensity and polarization distributions in the transverse plane for HVBs with *m* = − 1 and 3 represented on the Poincaré sphere. (**b**) The experimental polarization state and the intensity distribution of the scalar vector beams (I) *m* = − 1, (II) m = 3 and (III) their mixture obtained with the (i) oscillator and the (ii) high-power laser system.

On the other hand, if we introduce the SPPS into the optical system, the generation of HVBs is realized by an overlapping of the modes with topological charges *m* = 1 and *m* =  − 3. Their intensity and polarization distributions are illustrated in Fig. 4a. As in the previous case, scalar vortex modes are obtained for circular polarization. For a right-handed circular input polarization, the topological charge of the optical vortex is 1 (Fig. 4b column I) and for the inverted polarization state, *m* =  − 3 was achieved (Fig. 4b column II). A mixture of around 50% energy from each mode generates an HVB with an intensity profile depicted in Fig. 4b, Column III. The polarization state is characterized by a left-handed circular polarization around the singularity point determined by the polarization state of *m* = 1 mode which has the highest intensity near the optical axis. Farther from the axis, the local polarization passes from linear to elliptical with opposite handedness due to the influence of the vortex mode with *m* =  − 3.

**Figure 5:**
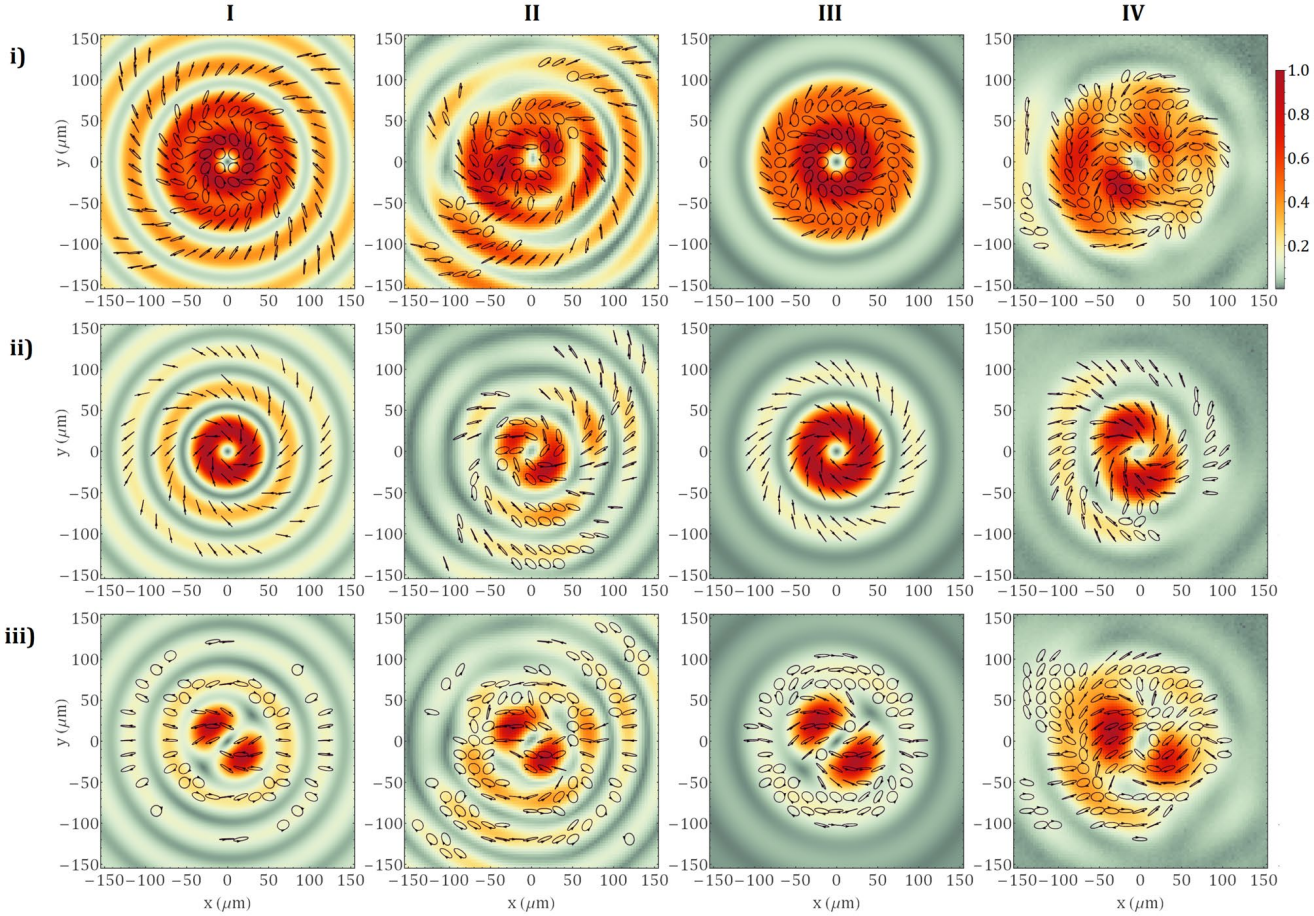
Theoretical modelling from the column I and experimental results from column II were obtained for a Sapphire crystal of 2.72 mm and an input beam generated by the laser oscillator. On the last two columns, III and IV, there are represented the theoretical and experimental results of the high-power laser system. The polarization of the input beam is (**i**) right-handed circular, (**ii**) left-handed circular and (**iii**) linear.

We will further investigate a more complex case when a Laguerre–Gauss beam is incident on the VBS comprising of a 2.72 mm thickness Sapphire crystal. For this value of the crystal thickness, the conversion efficiency of polarization is nearly *η* = 50% (Fig. 1b). Accordingly, 50% of the energy of the beam preserves its initial polarization state and the topological charge *m* =  − 1, and 50% will be a combination of *m* = 1 and *m* =  − 3 modes with a left, respectively right-circular polarization states. However, the weight of these two modes will depend on the input polarization state and can be 0 for some of the modes in the case of right or left-handed circular polarization. The generated focal spots presented in Fig. 5, column (I) and column (II) show the theoretical and experimental results using the oscillator beam while column (III) and column (IV) show the theoretical and experimental results using the pulsed, high-power laser system.

On row (i), Fig. 5,  are illustrated the intensity profile and the polarization map of the HVB attained by overlapping the modes *m* =  − 1 and − 3. One can say that the polarization follows the same tendency as in the case of the 5.44 mm thick Sapphire crystal, mentioned above. The only difference is given by the orientation of the polarization ellipses which rotate with 2*π* on a closed contour around the singularity for this case compared to 4*π* for the case shown in Fig. 4. The focal spot presents a more extended doughnut profile compared to that obtained by the superposition of two complex conjugated vortex modes, *m* =  ± 2. The VB with m =  ± 1 displayed on row (ii), has a “doughnut” intensity distribution specific to the topological charge. The local polarization state is linear. It is not similar to that for *m* =  ± 2 because the orientations of ellipses differ, from a topological charge value to another, due to the phase difference between the modes with right- and left-handed circular polarization. On row (iii) is highlighted a focal spot that has a two-lobe configuration. The HVB is a mixture of four vortex modes in the context in which the initial linear polarization is decomposed into right- and left-handed circular polarization. Thus, we will have two modes with *m* =  ± 1 with left-handed circular polarization and two modes with *m* =  − 1 and − 3 with right-handed circular polarization. The remarkable behaviour of breaking the circular symmetry of the intensity distribution is caused by the interference between the mode *m* =  − 3 and the mode *m* =  − 1, as their polarization states are not orthogonal, and the *m* =  − 3 mode acquires an additional amount *π* of Gouy phase due to the propagation to the focal plane.

To illustrate the effect of the Gouy phase, we present below a simplified mathematical model. We will consider that an operator $\mathcal{U}$ applied on a state representing the beam accounts for the propagation through the VBS. We are in the case where the conversion efficiency is *η* = 50%. We consider an initial beam with a diagonal polarization state at 45° between vertical and horizontal directions, identified by the state $\left|\mathrm{D}\rangle \right.$. The VHWP with *m* =  − 1 induces a vortex spatial phase ${e}^{-\mathrm{i }\theta }$. Thus, the incoming beam on the crystal is ${e}^{-\mathrm{i }\theta }\left| \mathrm{D }\rangle \right..$ We express this in the basis generated by the right/left circular polarization states for which the action of the operator $\mathcal{U}$ is known to be given by the matrix ${\mathbb{U}}$ in Eq. (5). We will consider that the operator $\mathcal{F}$ accounts for the propagation of the beam to the focal plane. Since we are not concerned with the spatial profile of the beam and how it changes from the lens to the focal plane, the operator $\mathcal{F}$ only adds the amount of the Gouy phase according to the topological charge of the mode. The initial state that was considered is given in Eq. (11). The effects of the operators $\mathcal{U}$ and $\mathcal{F}$ on the circularly polarized states are exemplified in Eq. (12). The propagation through the rest of the system is equivalent for all modes, thus it will be discarded.

11 $${e}^{-\mathrm{i }\theta }\left| \mathrm{D }\rangle \right.={e}^{-\mathrm{i }\theta }\left( \left| + \rangle \right.+\mathrm{i }\left| - \rangle \right. \right)/\sqrt{2}$$

12 $$\begin{aligned} \mathcal{U} {e}^{\mathrm{i }m \theta } \left| \pm \rangle \right. & =\left({e}^{\mathrm{i }m \theta }\left| \pm \rangle \right.+\mathrm{i} {e}^{\mathrm{i }\left(m\pm 2\right) \theta } \left| \mp \rangle \right.\right)/\sqrt{2} \\ \mathcal{F}{e}^{\mathrm{i }m \theta } \left| \pm \rangle \right. & ={e}^{\mathrm{i }m \theta +\mathrm{i }\frac{\pi }{2} \left(\left|m\right|+1\right)} \left| \pm \rangle \right. \end{aligned}$$

13 $$\begin{aligned} \mathcal{U} {e}^{-\mathrm{i }\theta }\left| \mathrm{D }\rangle \right. & =\left( \left| + \rangle \right.+\mathrm{i }\left| - \rangle \right.{e}^{2\mathrm{ i }\theta }\right){e}^{-\mathrm{i }\theta }/2+\mathrm{i }\left(\mathrm{i }\left| + \rangle \right.{e}^{-2\mathrm{ i }\theta }+\left| - \rangle \right.\right){e}^{-\mathrm{i }\theta }/2 \\ & =\left\{\left({e}^{\mathrm{i }\theta }-{e}^{-\mathrm{i }\theta }\right){e}^{-2\mathrm{ i }\theta }\left| + \rangle \right.+\mathrm{i }\left({e}^{\mathrm{i }\theta }+{e}^{-\mathrm{i }\theta }\right)\left| - \rangle \right.\right\}/2 \\ &=\mathrm{sin}\theta {e}^{-2\mathrm{ i }\theta }\left| + \rangle \right.+\mathrm{cos}\theta \left| -\rangle \right.\end{aligned}$$

14 $$\begin{aligned} \mathcal{F} \mathcal{U} {e}^{-\mathrm{i }\theta }\left| \mathrm{D }\rangle \right. & =\mathcal{F}\left( \left| + \rangle \right.{e}^{-\mathrm{i }\theta }+\mathrm{i }\left| - \rangle \right.{e}^{\mathrm{i }\theta }\right)/2+\mathrm{i }\mathcal{F}\left(\mathrm{i }\left| + \rangle \right.{e}^{-3\mathrm{ i }\theta }+\left| - \rangle \right.{e}^{-\mathrm{i }\theta }\right)/2 \\ & =\left( \left| + \rangle \right.{e}^{-\mathrm{i }\theta }+\mathrm{i }\left| - \rangle \right.{e}^{\mathrm{i }\theta }\right)/2+\left(\left| + \rangle \right.{e}^{-3\mathrm{ i }\theta }+\mathrm{i }\left| - \rangle \right.{e}^{-\mathrm{i }\theta }\right)/2 \\ & =\mathrm{cos}\theta {e}^{-2\mathrm{ i }\theta }\left|+\rangle \right.+\mathrm{icos}\theta \left|-\rangle \right.\end{aligned}$$

As we can infer from Eq. (13), the intensity of the beam after the uniaxial crystal does not depend on the azimuthal variable $\theta$, therefore the beam is circularly symmetric. This property can be checked considering an identical transverse intensity profile for all modes, after the crystal, by computing the squared absolute value of the state resulting from Eq. (13). Contrarily, the intensity of the beam, as given by the Eq. (14) depends on $\theta$ by square cos, which means that the additional π amount of phase acquired by the mode *m* =  − 3 explains the breaking of the circular symmetry. A simple theoretical analysis using the Debye-Wolf integral will show that a linear polarized beam strongly focused tends to produce an elliptical spot that lacks circular symmetry despite that the initial profile of the beam is circularly symmetric; for instance, see Fig. 3 in ref.^37^. The experimental work by Kotlyar et al.^38^, reported a similar phenomenon of circular symmetry breaking of the focal spot generated from a Gaussian beam with a very small waist radius (7$\lambda$), further focused with a subwavelength diffractive binary axicon. The focused beam also becomes more elliptical in shape with two lobes. However, for our system, we used a lens with a focal length $f=1\mathrm{m}$ and an exit pupil diameter of 20 mm. In this focusing regime, the contribution of the longitudinal electric field to the total intensity is less than 0.1%. From this argument, we can exclude the possibility that the observed effect is caused by the presence of the longitudinal electric field. Also, Eqs. (12–14) do not take into consideration the longitudinal field, yet an azimuthal dependence ${\left(\mathrm{cos}\theta \right)}^{2}$ of the intensity profile in the focal plane is predicted. This phenomenon is not dependent on the focusing regime.

## Conclusion

In conclusion, we investigated the intensity distribution and the polarization state of VVBs and HVBs in the focal plane of a lens. The beams were generated using a Sapphire crystal placed between two axicons. By changing the crystal thickness and the spatial distribution of the laser beam incident on the uniaxial crystal, the mode composition and their weights are varied. We have proven that we can control the diffraction patterns by controlling the polarization of the beam incident on the crystal. We have also shown that these are influenced by an additional Gouy phase difference acquired between the composing modes due to the propagation to the focal plane. The experimental results are in good agreement with the theory. The importance of the optical system is given by its versatility to be used in any laser power conditions (up to 33 GW @ 10 Hz, otherwise self-focusing of the laser beam into the first axicon and crystal is observed) as well as by reproducibility of the results.

## Methods

### Devices in the experimental setup

As laser sources, we used a Ti:Sapphire oscillator from FemtoLasers that operates in free-running regime at 780 nm and a high-power laser system (Legend Elite HE+) that provides laser pulses at 1 kHz repetition rate with a central wavelength λ = 800 nm and a pulse duration of 35 fs.

As we stated in the experimental setup subheading, the SPPS consists of a VHWP placed in between two QWPs. The VHWP is made from a Liquid Crystal Polymer (Thorlabs, Germany) for which the fast axis varies azimuthally. For this reason, the VHWP introduces a constant phase difference between the components of the optical field for which the polarization has an ordinary/extraordinary orientation. For example, in the case of the VHWP plate used in experiments, the polarization state of the resulting optical beam may be radial or azimuthal if the element is illuminated with a linearly polarized laser beam. It can be considered that VHWP independently modifies the components of the beam with right-circular polarization and left-circular polarization, realizing the exchange between the angular momentum due to the circular polarization and the angular momentum due to the phase structure of the beam (spin-angular momentum coupling^39,40^). A simple analysis using Jones matrices will reveal that illuminating the VHWP with circularly polarized beam results in a vortex beam with uniform polarization, orthogonal to the initial one. If the VHWP is inserted between quarter-wave plates, the components thus formed will transform a linearly polarized Gaussian beam into a linearly polarized Laguerre–Gauss beam with topological charge $\left|m\right|$ = 1. This method of producing vortex beams is advantageous for broadband beams; the VHWP is a true zero-order vortex plate.

The camera used in the experiment is a Basler acA4112-20um with a spatial resolution of 4096 × 3000 pixels. For the investigation of the polarization state, we have acquired images of the intensity profile in the focal plane of the lens *L*_4_ with the camera for each 6-degree rotation of the QWP from the PAS, more exactly from 0° to 180°. The images were used to determine the Stokes parameters for each pixel as described by Schaefer et al.^41^.
